# Quantifying Light Response of Leaf-Scale Water-Use Efficiency and Its Interrelationships With Photosynthesis and Stomatal Conductance in C_3_ and C_4_ Species

**DOI:** 10.3389/fpls.2020.00374

**Published:** 2020-04-24

**Authors:** Zi-Piao Ye, Yu Ling, Qiang Yu, Hong-Lang Duan, Hua-Jing Kang, Guo-Min Huang, Shi-Hua Duan, Xian-Mao Chen, Yu-Guo Liu, Shuang-Xi Zhou

**Affiliations:** ^1^Maths & Physics College, Jinggangshan University, Ji’an, China; ^2^College of Agricultural Sciences, Guangdong Ocean University, Zhanjiang, China; ^3^State Key Laboratory of Soil Erosion and Dryland Farming on the Loess Plateau, Northwest A&F University, Yangling, China; ^4^School of Life Sciences, University of Technology Sydney, Ultimo, NSW, Australia; ^5^College of Resources and Environment, University of Chinese Academy of Science, Beijing, China; ^6^Jiangxi Provincial Key Laboratory for Restoration of Degraded Ecosystems and Watershed Ecohydrology, Nanchang Institute of Technology, Nanchang, China; ^7^Department of Landscape Architecture, Wenzhou Vocational College of Science & Technology, Wenzhou, China; ^8^School of Life Sciences, Jinggangshan University, Ji’an, China; ^9^Soil Fertilizer and Environmental Resources Institute, Jiangxi Academy of Agricultural Sciences, Nanchang, China; ^10^Institute of Desertification Studies, Chinese Academy of Forestry, Beijing, China; ^11^The New Zealand Institute for Plant and Food Research Limited, Havelock North, New Zealand

**Keywords:** irradiance, leaf gas exchange, light response curve, maximum water use efficiency, model, plant functional type (PFT), saturation light intensity, transpiration

## Abstract

Light intensity (*I*) is the most dynamic and significant environmental variable affecting photosynthesis (*A*_n_), stomatal conductance (*g*_s_), transpiration (*T*_r_), and water-use efficiency (WUE). Currently, studies characterizing leaf-scale WUE–*I* responses are rare and key questions have not been answered. In particular, (1) What shape does the response function take? (2) Are there maximum intrinsic (WUE_i_; WUE_i–max_) and instantaneous WUE (WUE_inst_; WUE_inst–max_) at the corresponding saturation irradiances (*I*_i–sat_ and *I*_inst–sat_)? This study developed WUE_i_–*I* and WUE_inst_–*I* models sharing the same non-asymptotic function with previously published *A*_n_–*I* and *g*_s_–*I* models. Observation-modeling intercomparison was conducted for field-grown plants of soybean (C_3_) and grain amaranth (C_4_) to assess the robustness of our models versus the non-rectangular hyperbola models (NH models). Both types of models can reproduce WUE–*I* curves well over light-limited range. However, at light-saturated range, NH models overestimated WUE_i–max_ and WUE_inst–max_ and cannot return *I*_i–sat_ and *I*_inst–sat_ due to its asymptotic function. Moreover, NH models cannot describe the down-regulation of WUE induced by high light, on which our models described well. The results showed that WUE_i_ and WUE_inst_ increased rapidly within low range of *I*, driven by uncoupled photosynthesis and stomatal responsiveness. Initial response rapidity of WUE_i_ was higher than WUE_inst_ because the greatest increase of *A*_n_ and *T*_r_ occurred at low *g*_s_. C_4_ species showed higher WUE_i–max_ and WUE_inst–max_ than C_3_ species—at similar *I*_i–sat_ and *I*_inst–sat_. Our intercomparison highlighted larger discrepancy between WUE_i_–*I* and WUE_inst_–*I* responses in C_3_ than C_4_ species, quantitatively characterizing an important advantage of C_4_ photosynthetic pathway—higher *A*_n_ gain but lower *T*_r_ cost per unit of *g*_s_ change. Our models can accurately return the wealth of key quantities defining species-specific WUE–*I* responses—besides *A*_n_–*I* and *g*_s_–*I* responses. The key advantage is its robustness in characterizing these entangled responses over a wide *I* range from light-limited to light-inhibitory light intensities, through adopting the same analytical framework and the explicit and consistent definitions on these responses. Our models are of significance for physiologists and modelers—and also for breeders screening for genotypes concurrently achieving maximized photosynthesis and optimized WUE.

## Introduction

Stomata control the balance between carbon flux driven by photosynthesis and water flux dominated by transpiration, which is characterized by water-use efficiency (WUE) at various scales (Sinclair et al., 1984; Gilbert et al., 2011; Eamus et al., 2016; Medlyn et al., 2017). WUE can thus indicate the natural selection on the balance between these fluxes (Hetherington and Woodward, 2003). Characterizing the environmental impacts on WUE among plant species and/or plant function types can advance our knowledge on differential plant adaptation strategies, and improve our prediction on consequences of environmental challenges (Avola et al., 2008; Egea et al., 2011; Zhou et al., 2014, 2016; De Kauwe et al., 2015; Köhler et al., 2016; Ahrar et al., 2017). For instance, plant species with the highest WUE would show the greatest fitness in dry habitats (Dudley, 1996; Zhou et al., 2019). WUE is also an important metric in crop breeding and genotype selection, especially for irrigated crops whose water use significantly affects crop productivity and profitability (Duursma et al., 2013; Flexas et al., 2013; Bota et al., 2016; Webster et al., 2016).

WUE can be estimated using different techniques, based on observations of leaf gas exchange, stable isotope discrimination, and eddy covariance fluxes (Medlyn et al., 2017). Among these techniques, WUE is most commonly estimated by measuring leaf gas exchange, facilitated by portable photosynthesis system allowing simultaneous measurement of leaf-scale carbon and water fluxes (Medrano et al., 2015). WUE derived from leaf gas exchange measurement is usually defined as the ratio of net CO_2_ assimilation rate (*A*_n_) to stomatal conductance for water vapor (*g*_s_)—intrinsic water-use efficiency (WUE_i_; von Caemmerer and Farquhar, 1981), or the ratio of *A*_n_ to transpiration rate (*T*_r_)—instantaneous water-use efficiency (WUE_inst_; Fischer and Turner, 1978) (see Table 1 for a summary of parameters and units). WUE_i_ can be used to compare photosynthetic characteristics independently of evaporative demand (Linares and Camarero, 2012). WUE_inst_ is a key determinant of whole-plant WUE as it summarizes plant dry mass production per unit of water loss (Sinclair et al., 1984; Duursma et al., 2013; but see Medrano et al., 2015 for constraints). WUE_i_ and WUE_inst_ have been widely used as an index of plant and vegetation performances in response to various environmental changes, such as changed water or light availabilities, vapor pressure deficit (VPD), temperature and CO_2_ concentration (Aranda et al., 2007; Avola et al., 2008; Linares and Camarero, 2012; Duursma et al., 2013; Bota et al., 2016).

**TABLE 1:** List of major model parameters defining the light response curves of photosynthesis (*A*_n_), stomatal conductance (*g*_s_), intrinsic water use efficiency (WUE_i_), and instantaneous water use efficiency (WUE_inst_).

Symbol	Definition	Unit
*A*_n_	Net photosynthetic rate	μmol CO_2_ m^–2^ s^–1^
*A*_nmax_	Maximum net photosynthetic rate	μmol CO_2_ m^–2^ s^–1^
*g*_s_	Stomatal conductance	mol H_2_O m^–2^ s^–1^
*g*_s–max_	Maximum stomatal conductance	mol H_2_O m^–2^ s^–1^
*I*	Light intensity	μmol photons m^–2^ s^–1^
*I*_sat_	Saturation light intensity corresponding to maximum net photosynthetic rate	μmol photons m^–2^ s^–1^
*I*_g–sat_	Saturation light intensity corresponding to maximum stomatal conductance	μmol photons m^–2^ s^–1^
*I*_i–sat_	Saturation light intensity corresponding to maximum intrinsic water-use efficiency	μmol photons m^–2^ s^–1^
*I*_inst–sat_	Saturation light intensity corresponding to maximum instantaneous water-use efficiency	μmol photons m^–2^ s^–1^
*R*_d_	Mitochondrial CO_2_ release in the dark	μmol CO_2_ m^–2^ s^–1^
*T*_r_	Transpiration rate	mmol H_2_O m^–2^ s^–1^
WUE_i_	Intrinsic water-use efficiency	μmol CO_2_ mol^–1^ H_2_O
WUE_i–max_	Maximum intrinsic water-use efficiency	μmol CO_2_ mol^–1^ H_2_O
WUE_inst_	Instantaneous water-use efficiency	μmol CO_2_ mmol^–1^ H_2_O
WUE_inst–max_	Maximum instantaneous water-use efficiency	μmol CO_2_ mmol^–1^ H_2_O
α, α_0_, α_1_, α_2_	Initial slope of light response curve of *A*_n_, *g*_s_, WUE_i_ and WUE_inst_	mmol H_2_O m^–2^ s^–1^
β, β_0_, β_1_, β_2_	Inhibitor coefficient of light response curve of *A*_n_, *g*_s_, WUE_i_ and WUE_inst_	m^2^ s μmol^–1^ photons
γ, γ_0_, γ_1_, γ_2_	Saturation coefficient of light response curve of *A*_n_, *g*_s_, WUE_i_ and WUE_inst_	m^2^ s μmol^–1^ photons
*K*_i_	Residual intrinsic water-use efficiency	μmol CO_2_ mol^–1^ H_2_O
*K*_inst_	Residual instantaneous water-use efficiency	μmol CO_2_ mmol^–1^ H_2_O

Light is often viewed as the most significant environmental variable affecting photosynthesis, stomatal behavior and WUE (Knapp and Smith, 1987; Aranda et al., 2007; McAusland et al., 2016). Plants in most ecosystems experience rapid short-term variability in light resource (Smith et al., 1989), which can cause continual transition of *A*_n_, *g*_s_, *T*_r_, WUE_i_, and WUE_inst_ throughout the growing season (Knapp and Smith, 1990; Knapp, 1993). However, studies characterizing the light response of WUE are rare (McAusland et al., 2016). It is largely unknown whether there is a maximum WUE_i_ or WUE_inst_—and the corresponding saturation irradiance—for plants under dynamic irradiance conditions, or how plant species or plant function types (PFTs) would differ in their light responses of WUE_i_ and WUE_inst_.

Characterization of the interrelationships among light responses of *A*_n_, *g*_s_, *T*_r_, WUE_i_ and WUE_inst_—which can be simultaneously measured—will be fundamental to the scaling-up modeling of WUE–*I* responses at the whole-plant and ecosystem scale. The foremost step toward this direction calls for a robust model, with which (1) the WUE_i_ and WUE_inst_ responses to a gradient of irradiance intensity (*I*) levels (WUE_i_–*I* and WUE_inst_–*I* response curve, respectively) can be characterized, and (2) the key quantities defining the response curves—such as the initial slope of the response curve, the maximum WUE and the corresponding saturation irradiance—can be quantified. Ideally, the model can accurately represent the differential WUE_i_ and WUE_inst_ responses among plant species or PFTs, such as that reported between C_3_ and C_4_ species with contrasting light responses of photosynthesis, stomatal functioning, and WUE (Pearcy and Ehleringer, 1984; Knapp, 1993). For a given *A*_n_, *g*_s_ and *T*_r_ are higher in C_3_ than C_4_ plants, leading to higher WUE_i_ and WUE_inst_ in C_4_ plants, which has higher utilization efficiency of CO_2_ at relatively lower intercellular CO_2_ concentration (Pearcy and Ehleringer, 1984). The objectives of this study were to develop a leaf-scale WUE–*I* model and assess the model performance against experimental field observations of C_3_ and C_4_ species in order to answer key questions of how best to model the light response of WUE_i_ and WUE_inst_. In particular: (1) What shape does the leaf-scale WUE–*I* response function take? Is there a maximum WUE_i_ and/or WUE_inst_—and the corresponding saturation irradiances for plants under dynamic irradiance conditions? (2) Can the model well represent the differential WUE_i_–*I* and/or WUE_inst_–*I* response characteristics between C_3_ and C_4_ species? By integrating the published *A*_n_–*I* (Ye, 2007; Ye et al., 2013) and *g*_s_–*I* (Ye and Yu, 2008) response function, we developed an explicit WUE–*I* modeling framework and hypothesized that the species-specific light response curves of WUE_i_ and WUE_inst_ can be quantitatively characterized using the same non-asymptotic function. The hypothesis was tested using an observation-modeling intercomparison on WUE_i_–*I* and WUE_inst_–*I* responses for field-grown C_3_ [soybean (*Glycine max* L.)] and C_4_ species [grain amaranth (*Amaranthus hypochondriacus* L.)] under high *I* condition in the growing season. Model performance against that of the non-rectangular hyperbola model was also evaluated.

## Materials and Methods

### Analytical Models

A non-asymptotic model has been previously developed and tested to well characterize the light response of photosynthesis (Ye, 2007; Ye et al., 2013), with its simplified form as follows:

(1) $$A_{\text{n}}=\alpha \,\frac{1-\beta \,I}{1+\gamma \,I}\,{\text{I-R}}_{\text{d}}$$

where α is the initial slope of light response curve of photosynthesis, *I* is the irradiance, and β and γ are the photoinhibition coefficient and saturation coefficient, respectively, and *R*_d_ is the dark respiratory rate. The key model parameters are listed in Table 1.

The saturation irradiance (*I*_sat_) corresponding to the light-saturated photosynthetic rate (*A*_nmax_) can be calculated as follows:

(2) $$I_{\text{sat}}=\frac{\sqrt{\left(\beta +\gamma \right)/\beta }-1}{\gamma }$$

(3) $$A_{\text{nmax}}=\alpha \,{\left(\frac{\sqrt{\beta +\gamma }-\sqrt{\beta }}{\gamma }\right)}^{2}-R_{\text{d}}$$

Eq. 1 has been widely used to characterize photosynthetic light response curves of various plant species under different environmental conditions, highlighting its better performance than that of rectangular (Baly, 1935) and non-rectangular hyperbolic models (Thornley, 1976; Wargent et al., 2011; Xu et al., 2012a, b; Song et al., 2015; Chen et al., 2016). The rectangular and the non-rectangular hyperbolic models have been reported to overestimate *A*_nmax_ (dos Santos et al., 2013), and cannot quantify *I*_sat_ (Gomes et al., 2006; dos Santos et al., 2013; Song et al., 2015; Chen et al., 2016).

Meanwhile, a model of the same non-asymptotic form as Eq. 1 has been developed and tested to well characterize the light response of stomatal conductance (Ye and Yu, 2008), as follows:

(4) $$g_{\text{s}}=\alpha_{0}\,\frac{1-\beta_{0}\,I}{1+\gamma_{0}\,I}\,I+g_{\mathrm{s0}}$$

where α_0_ is the initial slope of light response curve of stomatal conductance, *g*_s__0_ is the residual stomatal conductance, and β_0_ and γ_0_ are two coefficients that are independent of *I* (Ye and Yu, 2008). Most existing stomatal conductance models cannot quantify the *g*_s–max_ or the corresponding *I*_g–sat_ under changing irradiance conditions (Dewar, 2002; Buckley et al., 2003; Buckley and Mott, 2013; Flexas et al., 2013). The *g*_s_–*I* model developed by Ye and Yu (2008) can well characterize the *g*_s_–*I* response, from which key parameters defining the *g*_s_–*I* response—such as *g*_s–max_ and *I*_g–sat_—can be easily obtained.

The saturation irradiance (*I*_g–sat_) corresponding to the light-saturated stomatal conductance (*g*_s–max_) can be calculated as follows:

(5) $$I_{g-\mathrm{sat}}=\frac{\sqrt{\left(\beta_{0}+\gamma_{0}\right)/\beta_{0}}-1}{\gamma_{0}}$$

(6) $$g_{\text{smax}}=\alpha_{0}\,{\left(\frac{\sqrt{\beta_{0}+\gamma_{0}}-\sqrt{\beta_{0}}}{\gamma_{0}}\right)}^{2}+g_{\mathrm{s0}}$$

Here, we hypothesize that the light response of WUE_i_ can be characterized using the same non-asymptotic form as that of the *A*_n_–*I* (Eq. 1) and *g*_s_–*I* (Eq. 4) response functions, as follows:

(7) $${\text{WUE}}_{\text{i}}=\alpha_{1}\,\frac{1-\beta_{1}\,I}{1+\gamma_{1}\,I}\,I-K_{i}$$

where α_1_ represents the initial slope of light response curve of WUE_i_, β_1_, and γ_1_ are coefficients that are independent of *I*, and *K*_i_ is the residual intrinsic water-use efficiency. The saturation irradiance (*I*_i–sat_) corresponding to the maximum WUE_i_ (WUE_i–max_) can be calculated as follows:

(8) $$I_{\text{i}-\text{sat}}=\frac{\sqrt{\left(\beta_{1}+\gamma_{1}\right)/\beta_{1}}-1}{\gamma_{1}}$$

(9) $${\text{WUE}}_{\text{i}-\text{max}}=\alpha_{1}\,{\left(\frac{\sqrt{\beta_{1}+\gamma_{1}}-\sqrt{\beta_{1}}}{\gamma_{1}}\right)}^{2}-K_{i}$$

Since *g*_s_ controls leaf *T*_r_ at a given *VPD* (Duursma et al., 2013), we hypothesize that the light response of WUE_inst_ can also be characterized using the same non-asymptotic function as that of WUE_i_–*I* response function (Eq. 7), as follows:

(10) $${\text{WUE}}_{\text{inst}}=\alpha_{2}\,\frac{1-\beta_{2}\,I}{1+\gamma_{2}\,I}\,I-K_{\text{inst}}$$

where α_2_ represents the initial slope of light response curve of WUE_inst_, β_2_ and γ_2_ are coefficients that are independent of *I*, and *K*_inst_ is the residual instantaneous water-use efficiency. The saturation irradiance (*I*_inst–sat_) corresponding to the maximum WUE_inst_ (WUE_inst–max_) can be calculated as follows:

(11) $$I_{\text{inst}-\text{sat}}=\frac{\sqrt{\left(\beta_{2}+\gamma_{2}\right)/\beta_{2}}-1}{\gamma_{2}}$$

(12) $${\text{WUE}}_{\text{inst}-\text{max}}=\alpha_{2}\,{\left(\frac{\sqrt{\beta_{2}+\gamma_{2}}-\sqrt{\beta_{2}}}{\gamma_{2}}\right)}^{2}-K_{\text{inst}}$$

In this study, we tested if Eqs. 7 and 10 can well characterize the species-specific WUE–*I* response characteristics against model-oriented field observations and the simulations using the non-rectangular hyperbola model—in terms of the initial slope of light response curve of WUE (α_1_ and α_2_, respectively), the maximum WUE_inst_ (WUE_i_ and WUE_inst–max_, respectively), and the saturation irradiance (*I*_i–sat_ and *I*_inst–sat_, respectively).

### Study Site and Plant Material

The field observations on one C_3_ species—soybean (*Glycine max* L.) and one C_4_ species—grain amaranth (*A. hypochondriacus* L.) were conducted at the Yucheng Comprehensive Experiment Station of the Chinese Academy of Sciences, located at the irrigation district of the Yellow River Basin in the North China Plain. This region is dominated by the warm-temperate semi-humid monsoon climate and is suitable for planting soybean and grain amaranth with high yields. This region has ample energy resource, and the light intensity in the growing season usually reaches ∼2000 μmol m^–2^ s^–1^ in sunny days. Soybean and grain amaranth were planted in field on May 3rd and June 15th 2012, respectively. All plants were kept under moist condition throughout the experiment.

### Light Response Curve Measurement

The leaf gas exchange measurements were conducted after 45 days of growth in field—June 16th for soybean and July 29th for grain amaranth. Fully expanded sun-exposed leaves of four plants for each species were measured using a portable photosynthesis system (LI-6400, Li-Cor Inc., Lincoln, NE, United States). Before each measurement, the leaf was acclimated in the chamber to achieve stable gas exchange, with reference CO_2_ concentration maintained at 380 μmol CO_2_ mol^–1^, irradiance intensity maintained at 2000 μmol photon m^–2^ s^–1^, and leaf temperature maintained at 35°C. After the leaf acclimated to the cuvette environment, the photosynthetic light response curve measurements were conducted with a descending gradient of irradiance intensity levels, as follows: 2000, 1800, 1600, 1400, 1200, 1000, 800, 600, 400, 200, 150, 100, 80, 50, and 0 μmol m^–2^ s^–1^. At each irradiance level, leaf gas exchange was monitored to ensure reaching steady-state plateau before data-logging. *VPD* was kept stable during measurements (Supplementary Figure S1). The *A*_n_–*I*, *g*_s_–*I*, WUE_i_–*I*, and WUE_inst_–*I* response curves were fitted by Eqs. 1, 4, 7, and 10, respectively. *I*_sat_, *I*_g–sat_, *I*_i–sat_, and *I*_inst–sat_ values were calculated following Eqs. 2, 5, 8, and 11, respectively. *A*_nmax_, *g*_s–max_, WUE_i–max_, and WUE_inst–max_ values were calculated following Eqs. 3, 6, 9, and 12, respectively.

### Data Analysis

All statistical tests were performed using the statistical package SPSS 18.5 statistical software (SPSS, Chicago, IL, United States). The analysis of variance (ANOVA) was used to assess species effects. Paired-sample *t* tests were conducted to test whether there were significant differences between fitted and measured values of quantitative traits (α, *A*_nmax_, *I*_sat_, α_0_, *g*_s–max_, *I*_g–sat_, α_1_, WUE_i–max_, *I*_i–sat_, α_2_, WUE_inst–max_, *I*_inst–sat_, etc.). Goodness of fit of the mathematical model to experimental observations was assessed using the coefficient of determination (*r*^2^ = 1–*SSE*/*SST*, where *SST* is the total sum of squares and *SSE* is the error sum of squares).

## Results

### Light Response Curves of *A*_n_, *g*_s_, and *T*_r_

**FIGURE 1:**
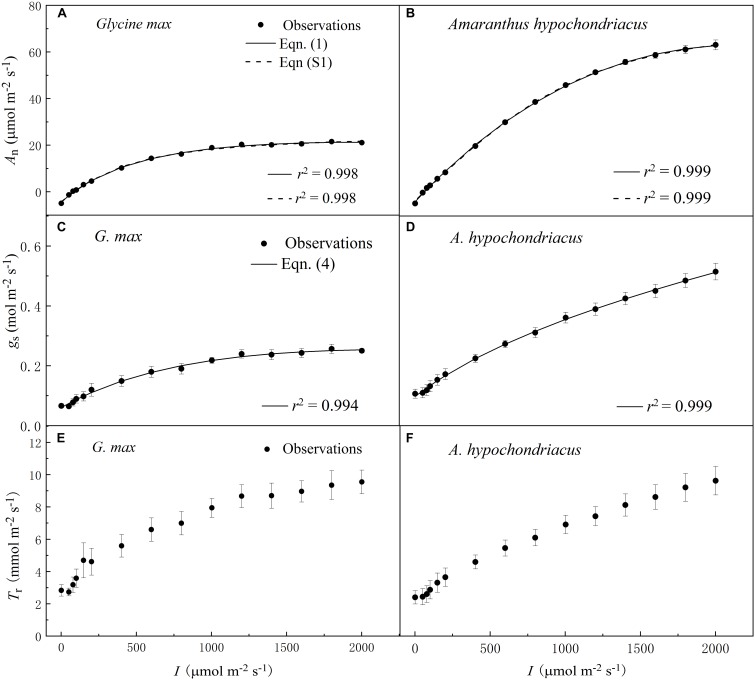
Irradiance (*I*) responses of net photosynthetic rate (*A*_n_) **(A,B)**, stomatal conductance (*g*_s_) **(C,D)** and transpiration rate (*T*_r_) **(E,F)** for C_3_ [soybean (*Glycine max*)] and C_4_ species [grain amaranth (*Amaranthus hypochondriacus*)], respectively. In plots **(A)** and **(B)**, solid lines were fitted using Eq. 1 and dashed lines were fitted using the non-rectangular hyperbola model (Eq. S1). In plots **(C)** and **(D)**, solid lines were fitted using Eq. 4. Data are the mean ± SE (*n* = 4).

The increase of *I* led to a rapid initial increase of *A*_n_ (Figures 1A,B), *g*_s_ (Figures 1C,D), and *T*_r_ (Figures 1E,F) for both C_3_ and C_4_ species. However, the initial increase rate of *A*_n_ was 100-fold higher than that of *g*_s_ for both species (Tables 2 and 3). The high coefficient of determination (*r*^2^) values indicated that the species-specific *A*_n_–*I* response curves fitted by Eq. 1—and the *g*_s_–*I* response curves fitted by Eq. 4—were highly representative of the observations for both species (Figure 1).

**TABLE 2:** Fitted (Eq. 1) and measured (Obs.) values of parameters defining the light response curve of photosynthesis for C_3_ (soybean) and C_4_ species (grain amaranth).

Species	α		*A*_nmax_ (μmol m^–2^ s^–1^)		*I*_sat_ (μmol m^–2^ s^–1^)		β (m^2^ s μmol^–1^)		γ (m^2^ s μmol^–1^)		*R*_d_ (μmol m^–2^ s^–1^)	
						
	Eq. 1	Obs.	Eq. 1	Obs.	Eq. 1	Obs.	Eq. 1	Obs.	Eq. 1	Obs.	Eq. 1	Obs.
Soybean	0.059 ± 0.005^a^	–	21.25 ± 0.53^b^	21.79 ± 0.58	1925.38 ± 60.30^a^	1800.00 ± 81.65	(1.20 ± 0.10) × 10^–4^	–	(1.25 ± 0.16) × 10^–3^	–	4.36 ± 0.46^a^	4.90 ± 0.29^a^
Grain amaranth	0.069 ± 0.001^a^	–	63.36 ± 2.46^a^	–	2186.67 ± 101.21^a^	–	(2.07 ± 0.15) × 10^–4^	–	(1.08 ± 0.29) × 10^–4^	–	4.18 ± 0.35^a^	4.99 ± 0.30^a^

*The parameters are initial slope of the *A*_*n*_–*I* curve (α), the maximum net photosynthetic rate (*A*_*nmax*_) and the corresponding saturation irradiance (*I*_*sat*_), photoinhibition coefficient (β), saturation coefficient (γ), and dark respiration rate (*R*_*d*_). All values are the means ± SE (*n* = 4). Different letters denote statistically significant differences (*P* ≤ 0.05) between soybean and grain amaranth within each column of fitted (Eq. 1) or measured (Obs.) values. There is no statistically significant difference between fitted (Eq. 1) and measured (Obs.) values for each parameter. See Table 1 for definitions of abbreviations.*

**TABLE 3:** Fitted (Eq. 4) and measured (Obs.) values of parameters defining the light response curve of stomatal conductance for C_3_ (soybean) and C_4_ species (grain amaranth).

Species	α_0_		*g*_s–max_ (mol m^–2^ s^–1^)		*I*_g__–__sat_ (μmol m^–2^ s^–1^)		β_0_ (m^2^ s μmol^–1^)		γ_0_ (m^2^ s μmol^–1^)		*g*_s__0_ (mol m^–2^ s^–1^)	
						
	Eq. 4	Obs.	Eq. 4	Obs.	Eq. 4	Obs.	Eq. 4	Obs.	Eq. 4	Obs.	Eq. 4	Obs.
Soybean	(3.5 ± 0.9) × 10^–4a^	–	0.26 ± 0.01	0.26 ± 0.02	2291.90 ± 259.17	1800.00 ± 81.65	(1.7 ± 0.7) × 10^–4^	–	(8.6 ± 5.5) × 10^–4^	–	0.06 ± 0.01^a^	0.07 ± 0.01^a^
Grain amaranth	(7.3 ± 3.0) × 10^–4a^	–	–	–	–	–	(-5.6 ± 6.1) × 10^–4^	–	(6.1 ± 5.5) × 10^–3^	–	0.09 ± 0.01^a^	0.11 ± 0.02^a^

*The parameters are initial slope of the *g*_*s*_–*I* curve (α_0_), coefficients β_0_ and γ_0_, the maximum stomatal conductance (*g*_*s–max*_) and the corresponding saturation irradiance (*I*_*g–sat*_), and the residual stomatal conductance (*g*_*s0*_). All values are the means ± SE (*n* = 4). Different letters denote statistically significant differences (*P* ≤ 0.05) between soybean and grain amaranth within each column of fitted (Eq. 4) or measured (Obs.) values. There is no statistically significant difference between fitted (Eq. 4) and measured (Obs.) values for each parameter. See Table 1 for definitions of abbreviations.*

Soybean exhibited a single-peaked pattern for both *A*_n_–*I* and *g*_s_–*I* responses, characterized by the increase of *A*_n_ and *g*_s_ with the increasing *I* until reaching the *A*_nmax_ and *g*_s–max_ at the corresponding *I*_sat_ and *I*_g–sat_, respectively (Figures 1A,C and Tables 2 and 3). Compared with Eq. 1, the non-rectangular hyperbola model (Supplementary Eq. S1) showed similarly high *r*^2^ value in simulating *A*_n_–*I* response curves but significantly overestimated the *A*_nmax_ (Figures 1A,B and Supplementary Table S1). Paired-sample *t* tests showed there were no significant differences between the fitted values and the measured values of *A*_nmax_, *I*_sat_, *g*_s–max_, and *I*_g–sat_ for soybean (Tables 2 and 3). Grain amaranth kept increasing its *A*_n_ and *g*_s_ within the range of irradiance intensity applied during measurements (0–2000 μmol photon m^–2^ s^–1^), without showing an observational *A*_nmax_, *I*_sat_, *g*_s–max_, or *I*_g–sat_ (Figures 1B,D and Tables 2 and 3). Grain amaranth showed relatively higher (not significant) initial increase rate of *g*_s_, characterized by an initial slope of the light response curve of *g*_s_ (α_0_) (Figure 1 and Tables 2 and 3).

### Light Response Curves of WUE_i_ and WUE_inst_

**FIGURE 2:**
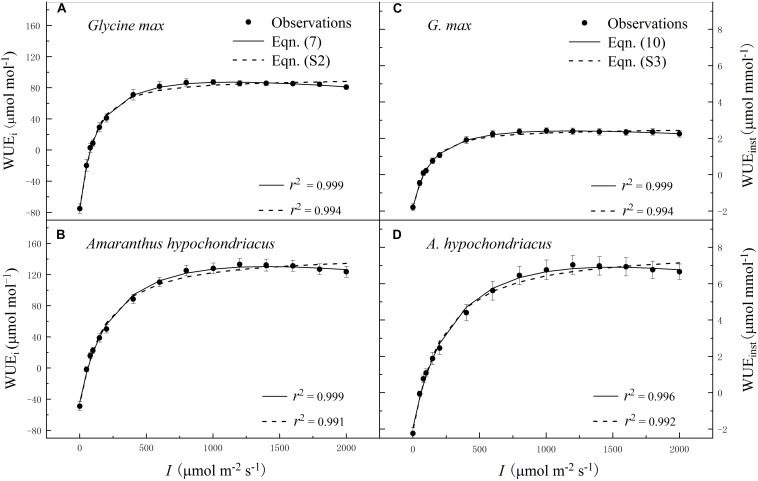
Irradiance (*I*) response of intrinsic water-use efficiency (WUE_i_) **(A,B)** and instantaneous water-use efficiency (WUE_inst_) **(C,D)** for C_3_ [soybean (*Glycine max*)] and C_4_ species [grain amaranth (*Amaranthus hypochondriacus*)], respectively. In plots **(A)** and **(B)**, solid lines were fitted using Eq. 7 and dashed lines were fitted using the non-rectangular hyperbola model (Eq. S2). In plots **(C)** and **(D)**, solid lines were fitted using Eq. 10 and dashed lines were fitted using the non-rectangular hyperbola model (Eq. S3). Data are the mean ± SE (*n* = 4).

Within the low range of irradiance intensity, WUE_i_ and WUE_inst_ of both species increased almost linearly with the increasing *I*. Both soybean and grain amaranth exhibited a single-peaked WUE_i_–*I* and WUE_inst_–*I* response pattern, respectively. In particular, both species showed an increase of WUE_i_ and WUE_inst_ with the increasing *I* until reaching the species-specific WUE_i–max_ and WUE_inst–max_ at the corresponding species-specific saturation irradiance levels (*I*_i–sat_ and *I*_inst–sat_, respectively) (Figure 2 and Tables 4 and 5). However, soybean showed significantly lower observed and fitted WUE_i–max_ and WUE_inst–max_ (*P* ≤ 0.05) than grain amaranth (Figure 2 and Tables 4 and 5). The two species showed no significant difference in *I*_i–sat_, *I*_inst–sat_ or the initial increase rate of WUE_i_ or WUE_inst_—characterized by a maximal slope of the light response curves (α_1_ and α_2_, respectively) (Figure 2 and Tables 4 and 5).

**TABLE 4:** Fitted (Eq. 7) and measured (Obs.) values of parameters defining the light response curve of intrinsic water-use efficiency for C_3_ (soybean) and C_4_ species (grain amaranth).

Species	α_1_		WUE_i–max_ (μmol mol^–1^)		*I*_i–__sat_ (μmol m^–2^ s^–1^)		β_1_ (m^2^ s μmol^–1^)		γ_1_ (m^2^ s μmol^–1^)		*K*_i_ (μmol mol^–1^)	
						
	Eq. 7	Obs.	Eq. 7	Obs.	Eq. 7	Obs.	Eq. 7	Obs.	Eq. 7	Obs.	Eq. 7	Obs.
Soybean	1.53 ± 0.25^a^	–	87.66 ± 3.38^b^	89.24 ± 3.26^b^	1153.95 ± 101.89^a^	1250.00 ± 262.99^a^	(8.49 ± 0.62) × 10^–5^	–	(7.52 ± 0.89) × 10^–3^	–	74.92 ± 6.16^a^	75.35 ± 5.98^a^
Grain amaranth	0.87 ± 0.19^a^	–	131.32 ± 7.83^a^	133.99 ± 7.63^a^	1417.60 ± 90.68^a^	1150.00 ± 125.83^a^	(1.21 ± 0.30) × 10^–4^	–	(3.72 ± 1.26) × 10^–3^	–	40.35 ± 4.24^b^	49.03 ± 5.69^b^

*The parameters are initial slope of the WUE_*i*_–*I* curve (α_1_), the maximum intrinsic water-use efficiency (WUE_*i–max*_) and the corresponding saturation irradiance (*I*_*i–sat*_), and coefficients β_1_ and γ_1_ and *K*_*i*_. All values are the means ± SE (*n* = 4). Different letters denote statistically significant differences (*P* ≤ 0.05) between soybean and grain amaranth within each column of fitted (Eq. 7) or measured (Obs.) values. There is no statistically significant difference between fitted (Eq. 7) and measured (Obs.) values for each parameter. See Table 1 for definitions of abbreviations.*

**TABLE 5:** Fitted (Eq. 10) and measured (Obs.) values of parameters defining the light response curve of instantaneous water-use efficiency for C_3_ (soybean) and C_4_ species (grain amaranth).

Species	α_2_		WUE_inst–max_ (μmol mmol^–1^)		*I*_inst–__sat_ (μmol m^–2^ s^–1^)		β_2_ (m^2^ s μmol^–1^)		γ_2_ (m^2^ s μmol^–1^)		*K*_inst_ (μmol mmol^–1^)	
						
	Eq. 10	Obs.	Eq. 10	Obs.	Eq. 10	Obs.	Eq. 10	Obs.	Eq. 10	Obs.	Eq. 10	Obs.
Soybean	0.035 ± 0.006^a^	–	2.42 ± 0.17^b^	2.47 ± 0.16^b^	1182.74 ± 63.01^a^	1300.00 ± 191.49^a^	(9.38 ± 1.29) × 10^–5^	–	(6.34 ± 0.82) × 10^–3^	–	1.78 ± 0.19^a^	1.80 ± 0.19^a^
Grain amaranth	0.037 ± 0.008^a^	–	6.99 ± 0.50^a^	7.03 ± 0.52^a^	1649.05 ± 260.38^a^	1300.00 ± 100.00^a^	(1.21 ± 0.32) × 10^–4^	–	(2.83 ± 0.83) × 10^–3^	–	1.81 ± 0.24^a^	2.24 ± 0.32^a^

*The parameters are initial slope of the WUE_*inst*_-*I* curve (α_2_), coefficients β_2_ and γ_2_ and *K*_*inst*_, the maximum instantaneous water-use efficiency (WUE_*inst–max*_), and the corresponding saturation irradiance (*I*_*inst–sat*_). All values are the means ± SE (*n* = 4). Different letters denote statistically significant differences (*P* ≤ 0.05) between soybean and grain amaranth within each column of fitted (Eq. 10) or measured (Obs.) values. There is no statistically significant difference between fitted (Eq. 10) and measured (Obs.) values for each parameter. See Table 1 for definitions of abbreviations.*

The high *r*^2^ values indicated that WUE_i_–*I* response curves fitted by Eq. 7—and the WUE_inst_–*I* response curves fitted by Eq. 10—were highly representative of the observations of both species (Figure 2 and Tables 4 and 5). There were no significant differences between fitted and observed values in WUE_i–max_, WUE_inst–max_, *I*_i–sat_, *I*_inst–sat_, *K*_i_, or *K*_inst_ (Tables 4 and 5). Compared with Eqs. 7 and 10, the non-rectangular hyperbola model (Supplementary Eqs. S2 and S3, respectively) showed similarly high *r*^2^ values but significantly overestimated WUE_i–max_ and WUE_inst–max_ for the two species (Supplementary Tables S2 and S3).

### Discussion

Our WUE_i_–*I* and WUE_inst_–*I* models represented cultivar-specific response curves over a wide range of light intensities extremely well (*r*^2^ ≥ 0.996), including the decline of WUE_i_ and WUE_inst_ beyond the saturation irradiances which the NH models cannot represent due to the asymptotic function. Our models can also return values for WUE_i–max_, WUE_inst–max_, *I*_i–sat_, and *I*_inst–sat_, which were in very close agreement with the measured values. The NH models cannot characterize the decline in WUE_i_ and WUE_inst_ induced by high light, leading to overestimations of WUE_i–max_ and WUE_inst–max_ (Supplementary Tables S2 and S3).

### Interrelationships of Light Responses of Photosynthesis, Stomatal Conductance, and Water-Use Efficiency

WUE_i_ and WUE_inst_ increased rapidly within low range of *I*, mainly driven by the uncoupled rapidity of photosynthetic and stomatal responses (Figures 1, 2 and Tables 4 and 5; Knapp and Smith, 1990; McAusland et al., 2016). In this study, both C_3_ and C_4_ species showed 100-fold higher initial increase rate of *A*_n_ (α) than that of *g*_s_ (α_0_) (Tables 2 and 3). The rapid initial increase of WUE_i_ and WUE_inst_—characterized by α_1_ and α_2_, respectively—occurred at low *g*_s_ (and at low *I*), when small increase in *g*_s_ exerted the greatest impacts on *A*_n_ and *T*_r_ (Hetherington and Woodward, 2003). The occurrence of the greatest *A*_n_ and *T*_r_ increase at low *g*_s_ also determined that α_1_ would be much higher than α_2_ for a given species (Figure 2 and Tables 4 and 5).

With the increasing *I* (from 0 to ∼800 μmol m^–2^ s^–1^), faster photosynthesis response than stomatal response led to the decline of intercellular CO_2_ concentration (*C*_i_) (Supplementary Figure S1; McAusland et al., 2016), causing further opening of stomatal pores (Mott, 1988) which allowed for diffusion of ambient CO_2_ into the leaf (Hetherington and Woodward, 2003). Further increase of *g*_s_—beyond the low *g*_s_ range—led to minimal increase of *A*_n_ and *T*_r_ (Hetherington and Woodward, 2003), such that WUE_i_ and WUE_inst_ flattened quickly after reaching the WUE_i–max_ and WUE_inst–max_ (Figure 2). Further increase of *I* beyond *I*_i–sat_ and *I*_inst–sat_ led to a decrease in WUE_i_ and WUE_inst_. To reach *A*_nmax_, both soybean and grain amaranth would have to show a decrease of WUE_i_ (or WUE_inst_) from WUE_i–max_ (or WUE_inst–max_) (Figures 1, 2).

### Differential Light Responses of Water-Use Efficiency Between C_3_ and C_4_ Species

The observation-modeling intercomparison in this study highlighted the differential single-peaked WUE_i_–*I* and WUE_inst_–*I* responses—besides differential *A*_n_–*I* and *g*_s_–*I* responses—between C_3_ and C_4_ species (Figure 2 and Tables 4 and 5). C_4_ species (grain amaranth) showed higher WUE_i_ and WUE_inst_ than C_3_ species (soybean), suggesting its better leaf-scale optimization of carbon uptake versus water loss than C_3_ species (Figures 1, 2 and Tables 2, 4, and 5). This may be due to higher photosynthetic capacity and rapidity of stomatal response (α_0_) in C_4_ species under changing irradiance conditions (Figure 1 and Tables 2 and 3), which facilitate relatively closer coupling between *A*_n_ and *g*_s_ in C_4_ species than C_3_ species (McAusland et al., 2016).

Moreover, this study identifies greater interspecific difference in WUE_inst_ than that in WUE_i_—at high *I* range when WUE_i_ and WUE_inst_ flatten (Figure 2 and Tables 4 and 5). C_3_ species (soybean) showed larger discrepancy between its WUE_i_–*I* and WUE_inst_–*I* responses than that of C_4_ species (grain amaranth). This may be due to differential water use strategies between C_3_ and C_4_ species—C_4_ species holds smaller *T*_r_ change per unit of *g*_s_ change in relative to C_3_ species (Knapp, 1993). These results quantitatively demonstrate that the differential WUE_i_–*I* responses between C_3_ and C_4_ species would not necessarily mirror their differential WUE_inst_–*I* responses (Figure 2).

These results support previous studies reporting that water conservation—in terms of high WUE—is an important consequence of the C_4_ photosynthetic pathway (besides high carbon gain rate) at different scales including single leaf, whole plant, and even whole communities (Ludlow and Wilson, 1972), contributing to the success of C_4_ species in high irradiance environments (Pearcy and Ehleringer, 1984; Knapp, 1993).

### Model Significance

By providing (1) analytical models characterizing the single-peaked light responses of WUE_i_ and WUE_inst_ and (2) key quantitative traits defining WUE_i_–*I* and WUE_inst_–*I* response differences between C_3_ and C_4_ species, this study provides a practical and robust modeling approach—in a form potentially applicable to WUE–*I* models at whole-plant and/or ecosystem scale. In particular, the key quantitative traits—the initial increase rates of WUE_i_ (α_1_) and WUE_inst_ (α_2_) besides that of *A*_n_ (α) and *g*_s_ (α_0_), the maximum WUE_i_ (WUE_i–max_) and WUE_inst_ (WUE_inst–max_) besides that of *A*_n_ (*A*_n__max_) and *g*_s_ (*g*_s–max_), and the corresponding saturation irradiances—will directly help physiologists and modelers investigate the interrelationships among photosynthesis, stomatal behavior, and WUE under changing irradiance conditions.

Meanwhile, the above quantitative traits allow for easier and more extensive evaluation of light-intensity consequences on carbon and water relations among different species and/or PFTs. Such quantitative information, gathered on a wider range of species and/or PFTs, could allow (1) a deeper understanding of interspecific variation in light response strategies (Knapp, 1993; Hetherington and Woodward, 2003; McAusland et al., 2016), and (2) a realistic representation of adaptive WUE–*I* response differences among PFTs into ecosystem modeling.

The explicit models developed in this study can be viewed as an initial step toward filling the gap between investigating the trends of interspecific variation in short-term leaf-scale WUE–*I* responses and translating the variation into improved process representation in models of plant and ecosystem scales. The findings in this study remain to be validated (1) with species of different growth form and PFT membership (e.g., slower-growing woody species), which could hold different light response strategies (Knapp and Smith, 1989), (2) with daily and seasonal integrals and/or whole-plant estimates of WUE that sometimes could show a low correlation with short-term leaf-scale WUE observations (Medrano et al., 2015), and (3) when leaf gas exchange is subjected to compound effects of other climatic conditions in current and future climate change scenarios.

## Conclusion

The newly developed models (Eqs. 7 and 10, respectively) allow robust reproduction of the differential single-peaked WUE_i_–*I* and WUE_inst_–*I* trends between C_3_ and C_4_ species and easy parameterization of key traits defining the trends (α_1_, *I*_i–sat_, *K*_i_ and WUE_i–max_, α_2_, *I*_inst–sat_, *K*_inst_, and WUE_inst–max_). The models can be employed for fast and accurate assessment of plant WUE_i_ and WUE_inst_ responses—besides that of photosynthetic and stomatal responses using a consistent modeling framework—across all light-limited, light-saturated, and photoinhibitory light intensities. These findings are useful (1) for breeders screening for ideal genotypes target with maximized photosynthesis capacity and optimized WUE, (2) for plant physiologists quantifying intra- and/or inter-specific variation in leaf-scale WUE–*I* responses, and (3) for modelers working on better representation of the coupling between carbon and water processes under dynamic irradiance conditions.

## Data Availability Statement

The datasets generated for this study are available on request to the corresponding author.

## Author Contributions

Z-PY and S-XZ drafted the work. All authors contributed substantially to the completion of this work and critically revised the work. Z-PY, H-JK, and Y-GL secured the funding.

## Conflict of Interest

The authors declare that the research was conducted in the absence of any commercial or financial relationships that could be construed as a potential conflict of interest.
