# Supplementary material for: Quantifying Light Response of Leaf-Scale Water-Use Efficiency and Its Interrelationships With Photosynthesis and Stomatal Conductance in C3 and C4 Species
Source: Front Plant Sci. 2020 Apr 24;11:374. doi: 10.3389/fpls.2020.00374 (PMC7199201; doi:10.3389/fpls.2020.00374)

**Quantifying Light Response of Leaf-scale Water-Use Efficiency and Its Interrelationships with Photosynthesis and Stomatal Conductance in C_3_ and C_4_ Species**

*Zi-Piao Ye^1#^, Yu Ling^2#^, Qiang Yu^3,4,5^, Hong-Lang Duan^6^, Hua-Jing Kang^7^, Guo-Min Huang^6^, Shi-Hua Duan^8^, Xian-Mao Chen^9^, Yu-Guo Liu^10*^ and Shuang-Xi Zhou^11*^*

*^1^Maths & Physics College, Jinggangshan University, Ji’an 343009, Jiangxi, China, ^2^College of Agricultural Sciences, Guangdong Ocean University, Zhanjiang 524088, Guangdong, China, ^3^State Key Laboratory of Soil Erosion and Dryland Farming on the Loess Plateau, Northwest A&F University , Yangling 712100, Shaanxi, China, ^4^School of Life Sciences, University of Technology Sydney, PO Box 123, Broadway, NSW 2007, Australia, ^5^College of Resources and Environment, University of Chinese Academy of Science, Beijing 100049, China, ^6^Jiangxi Provincial Key Laboratory for Restoration of Degraded Ecosystems and Watershed Ecohydrology, Nanchang Institute of Technology, Nanchang 330099, Jiangxi, China, ^7^Wenzhou Vocational College of Science & Technology, Wenzhou 325006, Zhejiang, China, ^8^School of Life Sciences, Jinggangshan University, Ji’an 343009, Jiangxi, China, ^9^Soil Fertilizer and Environmental Resources Institute, Jiangxi Academy of Agricultural Sciences, Nanchang 330200, Jiangxi, China, ^10^Institute of Desertification Studies, Chinese Academy of Forestry, Beijing 100091, China, ^11^The New Zealand Institute for Plant and Food Research Limited, Havelock North 4130, New Zealand*

*^*^For correspondence:*

[*shuangxi.zhou@plantandfood.co.nz*](mailto:shuangxi.zhou@plantandfood.co.nz)*;* [*liuyuguo@caf.ac.cn*](mailto:liuyuguo@caf.ac.cn)

^#^*These authors contributed equally to this work.*

**Supplementary Material**

The non-rectangular hyperbola model calculates the net CO_2_ assimilation rate as follows:

(S1)

where *A*_n_ is the net photosynthetic rate, *I* is light intensity, *A*_nmax_ is the maximum net photosynthetic rate, *α* is the initial slope of *A*_n_*-I* curves, *θ* is the convexity of curve, and *R*_d_ is dark respiration rate.

The non-rectangular hyperbolic model calculates the intrinsic water-use efficiency (WUE_i_) as follows:

(S2)

where WUE_i_ is the intrinsic water-use efficiency, *I* is light intensity, WUE_i-max_ is the maximum intrinsic water-use efficiency, *α*_1_ is the initial slope of WUE_i_–*I* curves, *θ* is the convexity of curve, *K*_i_ is the residual intrinsic water-use efficiency.

The non-rectangular hyperbolic model calculates the instantaneous water-use efficiency (WUE_inst_) as follows:

(S3)

where WUE_inst_ is the instantaneous water-use efficiency, *I* is light intensity, WUE_inst-max_ is the maximum instantaneous water-use efficiency, *α*_2_ is the initial slope of WUE_inst_–*I* curves, *θ* is the convexity of curve, *K*_inst_ is the residual instantaneous water-use efficiency.

**TABLE S1 | Fitted (Eqn. 1 and Eqn. S1) and measured values of the maximum net photosynthetic rate (*A*_nmax_) and the corresponding saturation irradiance (*I*_sat_) for soybean and grain amaranth.**

| **Species** | ***A*_nmax_ (μmol m^-2^ s^-1^)** | | | ***I*_sat_ (μmol m^-2^ s^-1^)** | | |
| --- | --- | --- | --- | --- | --- | --- |
|  | **Eqn. (1)** | **Eqn. (S1)** | **Obs.** | **Eqn. (1)** | **Eqn. (S1)** | **Obs.** |
| Soybean | 21.25 ± 0.53 ^b^ | 29.59 ± 1.39 ^a^ | 21.79 ± 0.58 ^b^ | 1925.38 ± 60.30 ^a^ | *–* | 1800.00 ± 81.65 ^a^ |
| Grain amaranth | 63.36 ± 2.46 ^b^ | 79.95 ± 5.00 ^a^ | *–* | 2186.67 ± 101.21 ^a^ | *–* | *–* |

*All values are the means ± SE (n = 4). Different letters denote statistically significant differences (P ≤ 0.05) – among the measured (Obs.) value, the value fitted by Eqn. (1) and the value fitted by the non-rectangular hyperbola model (Eqn. S1) – of the same parameter within each species.*

**TABLE S2 | Fitted (Eqn. 7 and Eqn. S2) and measured values of the maximum water-use efficiency (WUE_i-max_) and the corresponding saturation irradiance (*I*_i-sat_) for soybean and grain amaranth.**

| **Species** | **WUE_i-max_ (μmol mol^-1^)** | | | ***I*_i-sat_ (μmol m^-2^ s^-1^)** | | |
| --- | --- | --- | --- | --- | --- | --- |
|  | **Eqn. (7)** | **Eqn. (S2)** | **Obs.** | **Eqn. (7)** | **Eqn. (S2)** | **Obs.** |
| Soybean | 87.66 ± 3.38 ^b^ | 167.73 ± 7.04 ^a^ | 89.24 ± 3.26 ^b^ | 1153.92 ±101.89 ^a^ | *–* | 1250.00 ± 262.99 ^a^ |
| Grain amaranth | 131.32 ± 7.83 ^b^ | 182.24 ± 3.00 ^a^ | 133.99 ± 7.63 ^b^ | 1417.60 ± 90.68 ^a^ | *–* | 1150.00 ± 125.83 ^a^ |

*All values are the means ± SE (n = 4). Different letters denote statistically significant differences (P ≤ 0.05) – among the measured (Obs.) value, the value fitted by Eqn. (7) and the value fitted by the non-rectangular hyperbola model (Eqn. S2) – of the same parameter within each species.*

**TABLE S3 | Fitted (Eqn. 10 and Eqn. S3) and measured values of the maximum water-use efficiency (WUE_inst-max_) and corresponding saturation irradiance (*I*_inst-sat_) for soybean and grain amaranth.**

| **Species** | **WUE_inst-max_ (μmol mol^-1^)** | | | ***I*_inst-sat_ (μmol m^-2^ s^-1^)** | | |
| --- | --- | --- | --- | --- | --- | --- |
|  | **Eqn. (10)** | **Eqn. (S3)** | **Obs.** | **Eqn. (10)** | **Eqn. (S3)** | **Obs.** |
| Soybean | 2.42 ± 0.17 ^b^ | 4.34 ± 0.38 ^a^ | 2.47 ± 0.16 ^b^ | 1182.74 ± 63.01 ^a^ | *–* | 1300.00 ± 191.49 ^a^ |
| Grain amaranth | 6.99 ± 0.50 ^b^ | 9.63 ± 0.70 ^a^ | 7.03 ± 0.52 ^b^ | 1649.05 ± 260.38 ^a^ | *–* | 1300.00 ± 100.00 ^a^ |

*All values are the means ± SE (n = 4). Different letters denote statistically significant differences (P ≤ 0.05) – among the measured (Obs.) value, the value fitted by Eqn. (10) and the value fitted by the non-rectangular hyperbola model (Eqn. S3) – of the same parameter within each species.*

**FIGURE S1 |** **Vapor pressure deficit (VPD) (A, B) and intercellular CO_2_ concentration (*C*_i_) (C, D) over irradiance (*I*) levels for C_3_ [soybean (*Glycine max*)] and C_4_ species [grain amaranth (*Amaranthus hypochondriacus*)], respectively.** Data are the mean ± SE (*n* = 4).


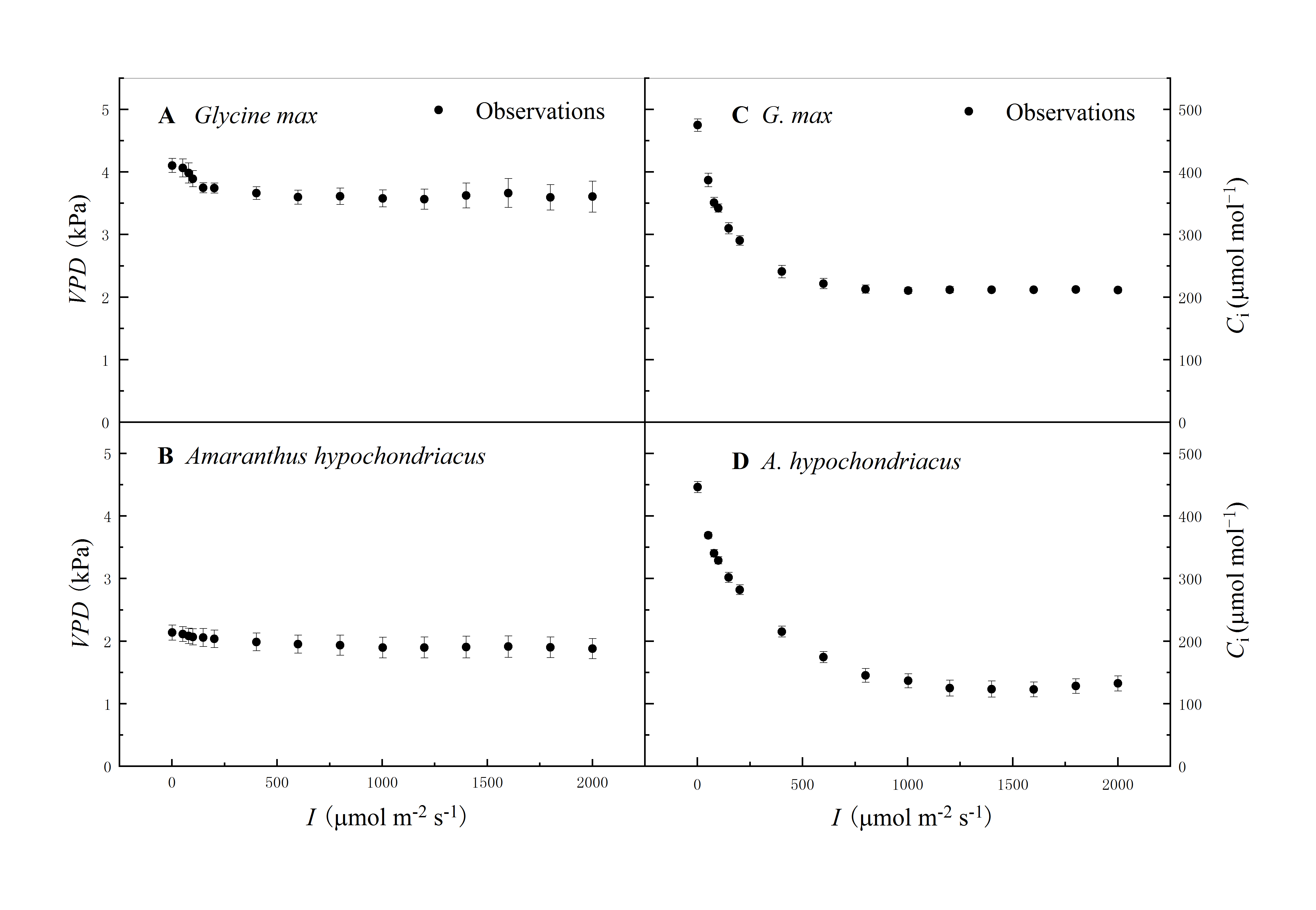

Supplement: Supplementary file 1 [file Data_Sheet_1.doc]
